# Supplementary figures and images for: An integrated anatomical, functional and evolutionary view of the Drosophila olfactory system
Source: EMBO Rep. 2025 May 19;26(12):3204–25. doi: 10.1038/s44319-025-00476-8 (PMC12187929; doi:10.1038/s44319-025-00476-8)

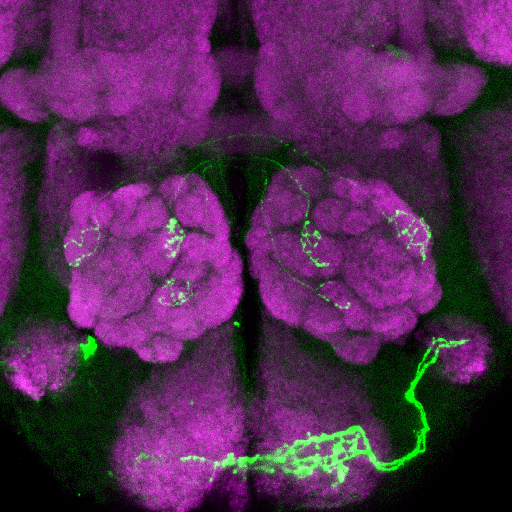

Supplement: Supplementary file 4 — Source data Fig. 2 [file 44319_2025_476_MOESM4_ESM.zip › Figure2G/Figure2G_elav_hsFLP_HS@P0_5min_3_b_composite_projection(z=1-37),g[11.74 60.72](RGB).tif]

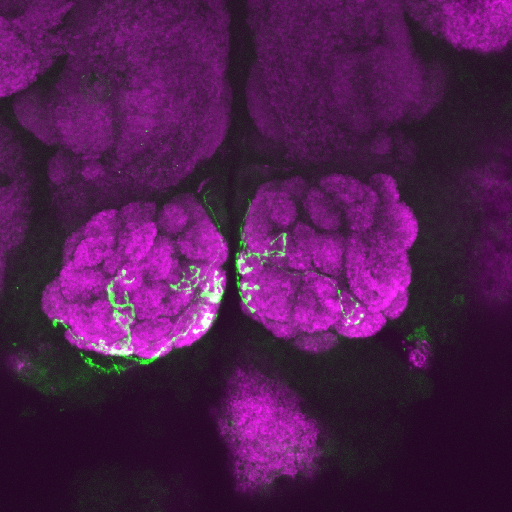

Supplement: Supplementary file 4 — Source data Fig. 2 [file 44319_2025_476_MOESM4_ESM.zip › Figure2H/Figure2H_elav_hsFLP_wt_HS@34hrB_5min_a_composite(z=1-25)_projection,g[1.1 65.0],m[-14.2 90.1](RGB).tif]
