# Supplementary material for: An integrated anatomical, functional and evolutionary view of the Drosophila olfactory system
Source: EMBO Rep. 2025 May 19;26(12):3204–25. doi: 10.1038/s44319-025-00476-8 (PMC12187929; doi:10.1038/s44319-025-00476-8)
Supplement: Supplementary file 6 — Expanded View Figures [file 44319_2025_476_MOESM6_ESM.pdf]

Expanded View Figures

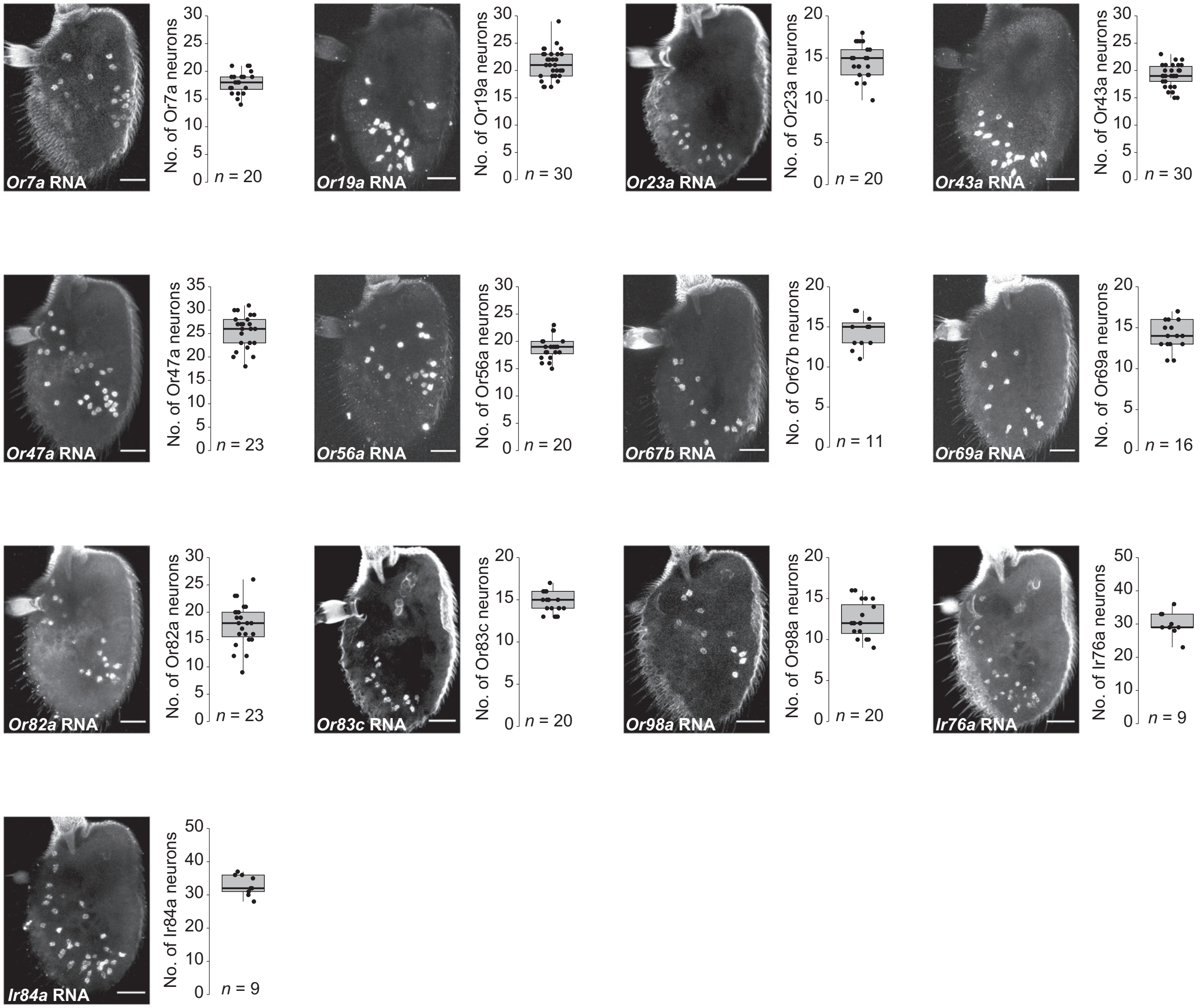

Figure EV1. Quantification of OSN populations.

Representative images of HCR RNA FISH on whole-mount antennae (control genotype *peb-Gal4*) using the indicated gene probes, and quantifications of OSN population size. Box plots show median (thick line) and first and third quartiles, while whiskers indicate data distribution limits, overlaid with individual data points. These data are reported in Dataset EV1. For Or69aA/B neurons, the image shown is with an *Or69aA* probe, but the quantifications are pooled from images using either *Or69aA* or *Or69aB* probes. For Ir76a neurons, we only counted the cells with strong signal, which likely correspond only to those in ac4 sensilla. *n* is indicated underneath each box plot. Scale bars, 25 µm.

 © The Author(s)

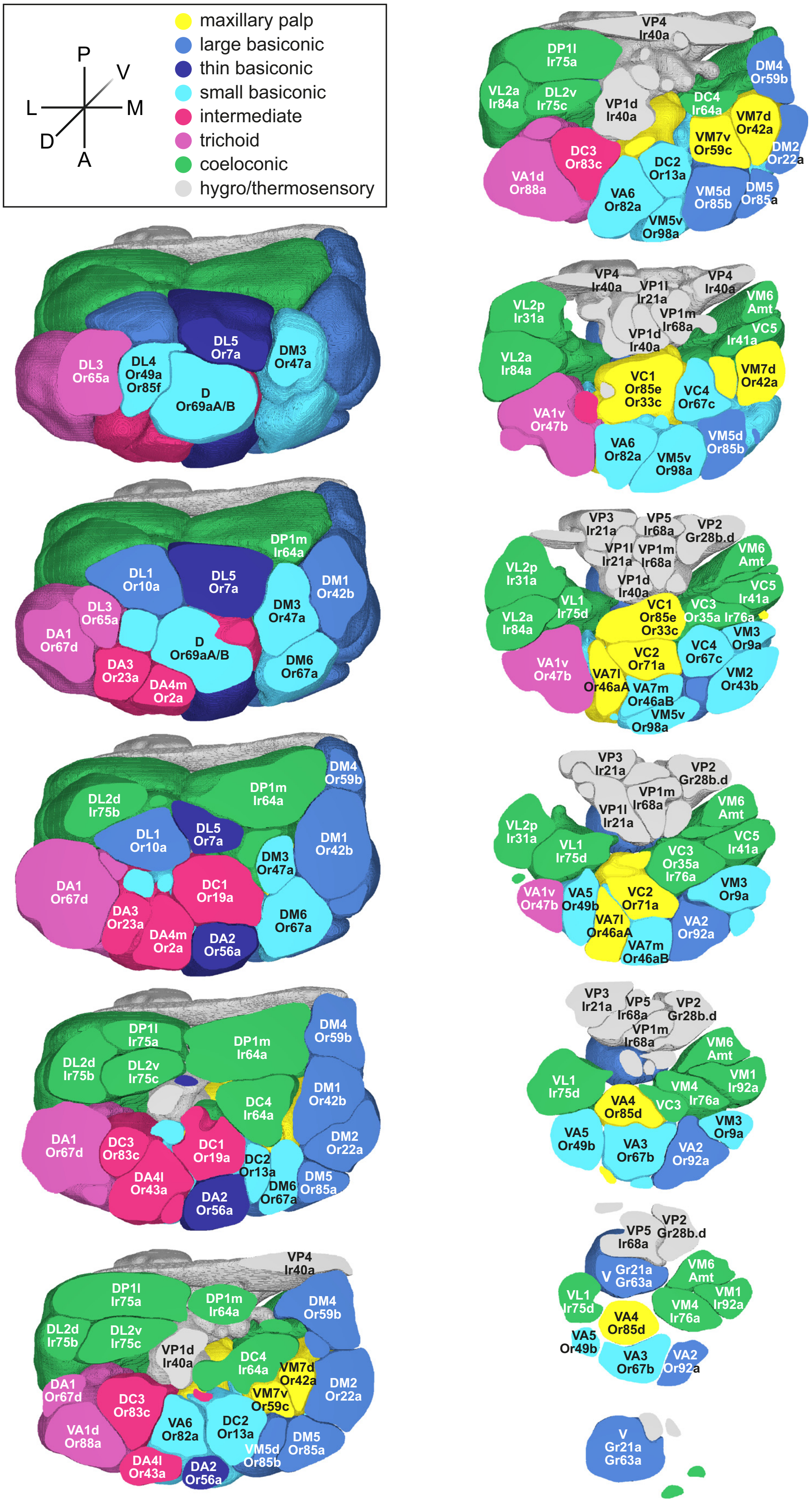
maxillary palp
large basiconic
thin basiconic
small basiconic
intermediate
trichoid
coeloconic
hygro/thermosensory
P
V
L
M
D
A
DL3 Or65a
DL4 Or49a Or85f
DL5 Or7a
DM3 Or47a
D Or69aA/B
DP1m Ir64a
DL1 Or10a
DM1 Or42b
DA1 Or67d
DA3 Or23a
DA4m Or2a
DM6 Or67a
DM4 Or59b
DL2d Ir75b
DC1 Or19a
DA2 Or56a
DP1l Ir75a
DL2v Ir75c
DC4 Ir64a
DM2 Or22a
DC3 Or83c
DA4l Or43a
DC2 Or13a
DM5 Or85a
VP4 Ir40a
VP1d Ir40a
VM7d Or42a
VM7v Or59c
VA1d Or88a
VA6 Or82a
VM5d Or85b
VM5v Or98a
VL2a Ir84a
VP1l Ir21a
VP1m Ir68a
VL2p Ir31a
VM6 Amt
VC5 Ir41a
VC1 Or85e Or33c
VC4 Or67c
VA1v Or47b
VP3 Ir21a
VP5 Ir68a
VP2 Gr28b.d
VL1 Ir75d
VC3 Or35a Ir76a
VM3 Or9a
VC2 Or71a
VA7l Or46aA
VA7m Or46aB
VM2 Or43b
VA5 Or49b
VA2 Or92a
VM4 Ir76a
VM1 Ir92a
VA4 Or85d
VA3 Or67b
V Gr21a Gr63a

◀ **Figure EV2. Antennal lobe atlas of transverse sections.**

Transverse sections along the dorsal-ventral axis of an updated antennal lobe atlas, with coloring as in Fig. 5 and Dataset EV2. Such views are more typical of those obtained during in vivo calcium imaging experiments.

 © The Author(s)
